# Plant Responses to Radionuclides and Heavy Metals in Uranium Mining Sites: Mechanisms and Phytoremediation

**DOI:** 10.3390/biology15161382

**Published:** 2026-08-13

**Authors:** Madina Kairullova, Meirat Bakhtin, Kuralay Ilbekova, Danara Ibrayeva

**Affiliations:** Scientific Research Institute of Radiobiology and Radiation Protection, Astana Medical University, Astana 010000, Kazakhstan

**Keywords:** uranium mining, radionuclides, heavy metals, plants, phytoremediation, ecological monitoring

## Abstract

Uranium mining and processing have resulted in widespread environmental contamination by radionuclides and associated heavy metals, creating long-term ecological challenges because of their persistence, mobility, and bioavailability. The aim of this review is to evaluate knowledge on the uptake, accumulation, and biological effects of radionuclides and associated heavy metals in plants, identify the environmental factors governing their bioavailability, and assess recent advances in phytoremediation strategies for the sustainable restoration of uranium-contaminated ecosystems. A critical analysis of the published literature was conducted to evaluate contaminant sources, environmental factors regulating bioavailability, root and foliar uptake pathways, internal transport mechanisms, morphological, physiological, and biochemical biomarkers of plant stress, and phytoremediation approaches. The reviewed evidence demonstrates that plant responses occur at multiple levels of biological organization, including alterations in growth and anatomy, disturbances in photosynthesis and nutrient metabolism, and activation of antioxidant defense systems. Integrating these biomarkers provides a more comprehensive assessment of contaminant-induced stress than individual indicators alone. The literature further indicates that phytostabilization and phytoextraction, particularly when combined with soil amendments and beneficial rhizosphere microorganisms, represent promising approaches for reducing contaminant mobility and supporting ecosystem restoration. However, important knowledge gaps remain regarding native plant species and long-term field validation in uranium mining regions. This review provides a scientific basis for improving ecological monitoring, environmental risk assessment, and sustainable rehabilitation of uranium-contaminated ecosystems.

## Simple Summary

Uranium mining and processing generate long-term contamination by radionuclides and associated heavy metals that threaten soil quality, vegetation, and ecosystem functioning. Plants are continuously exposed to these contaminants and develop morphological, physiological, and biochemical responses that can serve as sensitive indicators of environmental stress. The aim of this review is to evaluate knowledge on the uptake, accumulation, and biological effects of radionuclides and associated heavy metals in plants, identify the environmental factors governing their bioavailability, and assess recent advances in phytoremediation strategies for the sustainable restoration of uranium-contaminated ecosystems. This review summarizes current knowledge on the ecological impacts of uranium mining on vegetation, with particular emphasis on plant morphophysiological and biochemical responses, bioindicator potential, and phytoremediation strategies. Special attention is given to studies conducted in uranium mining regions worldwide, highlighting mechanisms of plant adaptation to combined radiological and heavy metal stress and identifying future research priorities for the ecological restoration of contaminated mining landscapes.

## 1. Introduction

Uranium mining and processing are among the most significant anthropogenic sources of environmental contamination, resulting in the long-term release of radionuclides and associated heavy metals into terrestrial ecosystems [1]. Mining operations, ore processing, and tailings disposal generate complex mixtures of contaminants, including uranium, arsenic, molybdenum, lead, cadmium, and other potentially toxic elements that accumulate in soils and persist for decades after mine closure [2,3]. These contaminants alter the physicochemical properties of soils, impair nutrient cycling, reduce microbial activity, and ultimately disrupt ecosystem functioning and vegetation development [2,4,5].

In particular, mine tailings are finely ground particles that exhibit extreme geochemical properties (pH, salinity, compaction) and contain high levels of toxic metals and metalloids, making them inherently inhibitory to the colonisation of plants [6].

Plants are continuously exposed to this combination of radiological and chemical stressors and therefore represent one of the most sensitive biological components of contaminated ecosystems. As primary producers, they regulate the migration of radionuclides and heavy metals through the soil–plant system and constitute an important pathway for the transfer of contaminants to higher trophic levels [7]. At the same time, plant communities integrate the cumulative effects of environmental stress over long periods, making them reliable indicators of ecosystem degradation and recovery following mining activities [8,9,10].

Long-term exposure to radionuclides and heavy metals induces profound changes in plant community structure and ecosystem succession. Sensitive native species are gradually replaced by stress-tolerant ruderal and pioneer species, resulting in reduced biodiversity and simplified vegetation communities [11,12]. The establishment of stable vegetation on mine wastes depends on multiple environmental factors, including residual radioactivity, soil physicochemical properties, nutrient availability, and regional climatic conditions [10,11]. Consequently, the composition and productivity of vegetation have become important indicators for evaluating ecosystem health and the effectiveness of mine reclamation programs.

Although numerous studies have documented radionuclide contamination in soils, water, and sediments surrounding uranium mines, considerably less attention has been devoted to understanding the adaptive responses of plants to chronic combined exposure to ionizing radiation and heavy metals [4,10]. Available evidence indicates that plant species differ markedly in their ability to absorb, translocate, and tolerate uranium. While some species exhibit hyperaccumulation potential, others effectively immobilize uranium within root tissues, thereby restricting its transport to aerial organs. Environmental factors such as soil pH, organic matter content, redox conditions, and metal speciation further regulate uranium bioavailability and plant uptake [13,14].

These plant-specific responses are reflected in a wide range of morphological, physiological, and biochemical alterations that can serve as sensitive biomarkers of environmental stress. Simultaneously, the ability of certain plant species to stabilize or extract uranium from contaminated soils has attracted increasing attention due to its potential applications in phytoremediation. Understanding the relationships between contaminant availability, plant adaptation, and phytoremediation efficiency is therefore essential for ecological risk assessment and the development of sustainable rehabilitation strategies for uranium mining regions [9,15].

The added value of this review lies in its integrated approach to understanding plant responses to radionuclides and associated heavy metals in uranium mining environments. The review brings together current knowledge on contaminant bioavailability, plant uptake and transport, morphological, physiological, biochemical, and molecular responses, plant resistance mechanisms, and phytoremediation strategies within a single conceptual framework. This integrated perspective provides a comprehensive synthesis of current knowledge, identifies existing research gaps, and highlights future research directions for environmental monitoring, ecological risk assessment, and the sustainable restoration of contaminated ecosystems.

The aim of this review is to evaluate knowledge on the uptake, accumulation, and biological effects of radionuclides and associated heavy metals in plants, identify the environmental factors governing their bioavailability, and assess recent advances in phytoremediation strategies for the sustainable restoration of uranium-contaminated ecosystems.

This review is organized into thematic sections covering the sources and environmental pathways of radionuclides and associated heavy metals, factors controlling their bioavailability, mechanisms of plant uptake, transport, and accumulation, morphological, physiological, biochemical, and resistance responses of plants, and current phytoremediation strategies for uranium-contaminated environments. The final sections discuss current research gaps, future perspectives, and the main conclusions of the review.

### Literature Search Strategy

**Figure 1:**
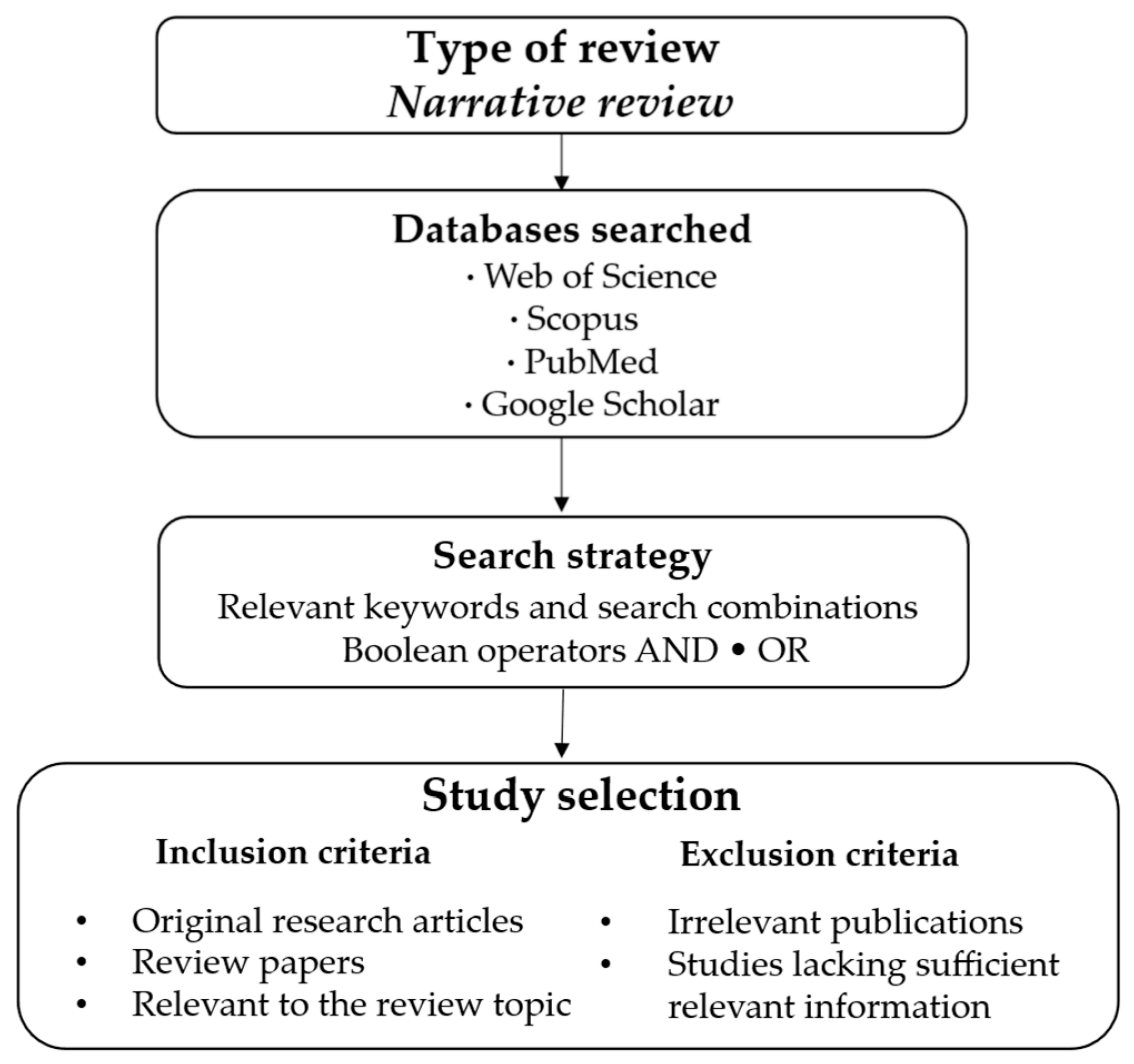
Flowchart of the literature search strategy and study selection process.

This review is a narrative review of the published literature on plant responses to radionuclides and associated heavy metals. The literature search was conducted using the Web of Science, Scopus, PubMed, and Google Scholar databases. Relevant keywords and their combinations were applied using the Boolean operators AND and OR. Original research articles and review papers relevant to the review topic were included, whereas irrelevant publications and studies lacking sufficient relevant information were excluded. The literature search and study selection strategy are summarized in Figure 1.

## 2. Sources and Pathways of Radionuclides and Heavy Metals Entering the Soil–Plant System

Sources of radionuclides and associated heavy metals entering soils can be broadly classified into natural and anthropogenic categories [3]. Natural sources include the weathering of uranium-bearing rocks, volcanic activity, and the natural radioactive decay of geological materials. Anthropogenic sources are primarily associated with uranium mining and processing activities, including ore extraction, crushing, hydrometallurgical processing, waste rock disposal, in situ leaching, tailings storage, mine drainage, process effluents, dust emissions, and radon exhalation. In uranium mining regions, anthropogenic sources represent the dominant contributors to soil contamination because of the large-scale release and long-term persistence of radionuclides and associated heavy metals [2,3,16].

Throughout the mining lifecycle, including ore extraction, crushing, hydrometallurgical processing, waste rock disposal, in situ leaching, and long-term storage of tailings, substantial quantities of radionuclides and associated potentially toxic elements are released into the environment [17]. These activities contribute to the contamination of soils, surface and groundwater, and the atmosphere through mine drainage, process effluents, seepage from tailings, dust emissions, and radon exhalation [17,18,19].

The principal contaminants include uranium-series radionuclides (^238^U, ^234^U, ^226^Ra, ^222^Rn, ^210^Pb), thorium isotopes, and associated heavy metals and metalloids such as As, Pb, Cd, Mo, Cu, Zn, Ni, Co, Cr, and Mn [20,21]. Numerous studies have demonstrated elevated concentrations of these elements in soils, sediments, waters, and vegetation surrounding uranium mining and processing facilities, indicating their long-term persistence and migration within terrestrial ecosystems [20,21,22].

However, the ecological impact of these contaminants is determined not only by their total concentration but also by their chemical speciation, mobility, and bioavailability. These characteristics govern the transfer of radionuclides and heavy metals into the soil–plant system and ultimately determine their uptake, accumulation, and toxicity in plants [23,24]. Therefore, the chemical speciation and bioavailability of radionuclides and heavy metals are key factors determining their mobility in soils, uptake by plants, and subsequent accumulation in plant tissues.

## 3. Forms of Contaminants in Soils and Factors Affecting Their Bioavailability

Biological availability of radionuclides and heavy metals is one of the key factors determining their ecological risk and subsequent uptake by plants. Unlike total elemental concentrations in soils, bioavailability refers to the fraction of contaminants that can be released into the soil solution and become available for absorption by plant roots. Consequently, the mobility of contaminants largely governs their migration through the soil–plant system and their transfer into terrestrial food chains [25].

In uranium mining regions, radionuclides and associated heavy metals occur in various geochemical forms that differ markedly in their mobility and availability to plants. These include dissolved ions in the soil solution, exchangeable fractions, species adsorbed onto clay minerals and iron, aluminum, and manganese oxides, complexes bound to soil organic matter, and elements incorporated into primary and secondary minerals. Among these fractions, dissolved and exchangeable forms are considered the most bioavailable, whereas contaminants strongly associated with the mineral matrix generally exhibit low mobility and limited plant availability [13].

**Figure 2:**
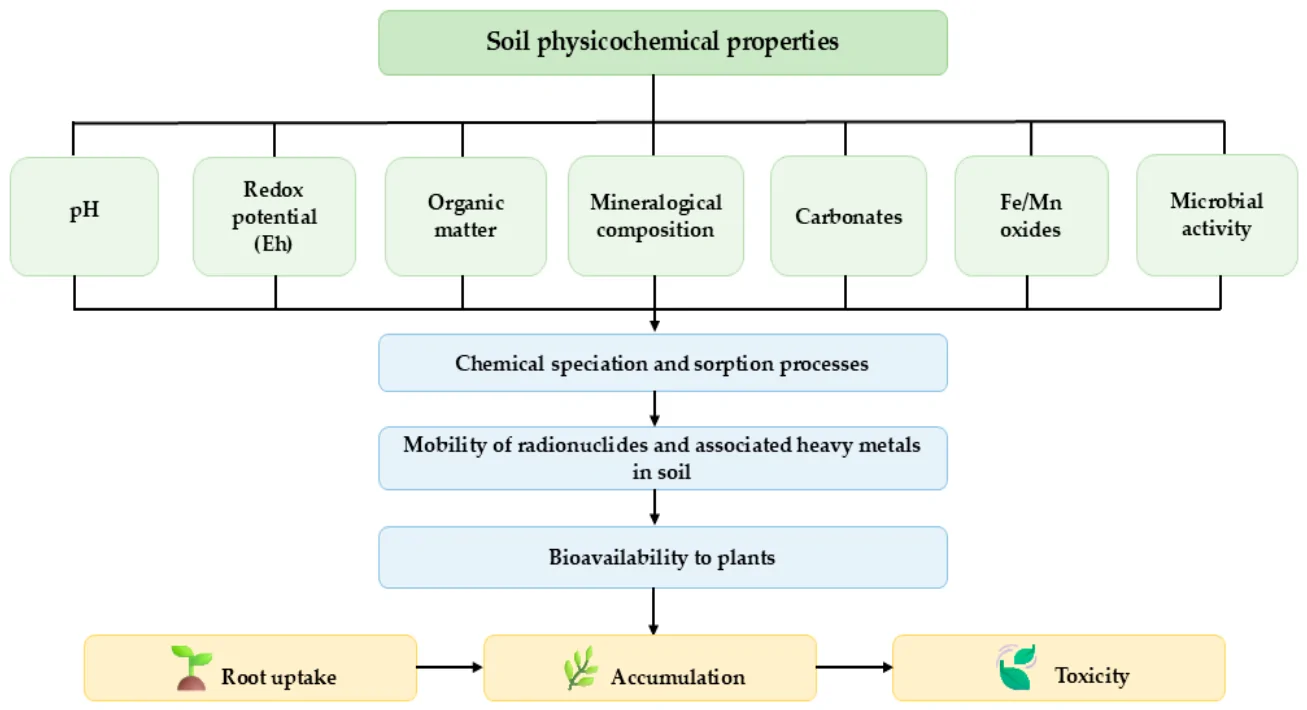
Influence of soil physicochemical properties on the chemical speciation, mobility, bioavailability, and plant uptake of radionuclides and associated heavy metals in the soil–plant system.

The distribution of contaminants among these geochemical fractions is controlled by several soil physicochemical properties, including pH, redox potential (Eh), organic matter content, mineralogical composition, carbonate concentration, and microbial activity [26], which collectively regulate contaminant mobility and bioavailability (Figure 2).

Soil pH is one of the principal factors regulating contaminant mobility. Acidic conditions promote the desorption of cationic metals such as Cd, Pb, Ni, Zn, and Cu from soil particles and enhance the dissolution of carbonate and hydroxide minerals, increasing their concentrations in the soil solution and facilitating plant uptake. Conversely, under neutral or alkaline conditions, heavy metals are more effectively retained by mineral phases and soil organic matter, thereby reducing their mobility and bioavailability [25].

For uranium, redox conditions play a decisive role in determining its chemical speciation. Under reducing conditions, uranium predominantly occurs as tetravalent U(IV), forming sparingly soluble minerals such as uraninite (UO_2_). In contrast, oxidizing environments favor the formation of hexavalent uranium U(VI), mainly present as the uranyl ion (UO_2_^2+^), which readily forms soluble complexes with carbonates, phosphates, and organic ligands. The formation of uranyl–carbonate complexes substantially increases uranium mobility, particularly in well-aerated calcareous and arid soils [27,28].

Soil organic matter also strongly influences contaminant behavior. Humic and fulvic substances may immobilize radionuclides and heavy metals by complexation, thereby reducing the concentration of free ions in the soil solution. However, soluble organo-metallic complexes can also enhance the mobility of certain elements, particularly uranium, copper, and nickel, facilitating their migration within the soil profile [26]. Similarly, iron and manganese oxides act as important sorbents for uranium, radium, lead, arsenic, and other elements. Changes in pH or redox conditions may dissolve these oxide phases and release previously immobilized contaminants, thereby increasing their bioavailability [13].

Soils affected by uranium mining are typically characterized by elevated concentrations of naturally occurring radionuclides (^238^U, ^232^Th, and ^226^Ra), associated heavy metals, and altered physicochemical properties. Mining activities disturb soil structure, reduce organic carbon content, and increase the proportion of fine mineral particles, thereby modifying contaminant mobility. Investigations conducted in abandoned uranium mining areas of Spain and China have demonstrated elevated concentrations of uranium and its decay products together with increased proportions of mobile contaminant fractions, resulting in enhanced migration within the soil–plant system [29,30].

Therefore, the ecological risk posed by radionuclides and heavy metals depends not only on their total concentrations in soils but primarily on their chemical speciation and the environmental factors controlling their bioavailability. These processes ultimately determine contaminant uptake, accumulation, and toxicity in plants.

### Physicochemical Reactions and Metal Interactions Governing Plant Uptake

The uptake of radionuclides and heavy metals by plants is governed by physicochemical processes that determine contaminant speciation, solubility, sorption, and mobility in the rhizosphere. Because only dissolved species present in the soil solution are readily available for root uptake, changes in chemical speciation directly influence contaminant bioavailability and subsequent accumulation in plant tissues [31,32].

Among radionuclides, uranium exhibits one of the strongest dependencies on environmental conditions. Under oxidizing conditions, uranium predominantly occurs as the uranyl ion (UO_2_^2+^), whereas reducing conditions favor the formation of sparingly soluble UO_2_ [1,31]. In carbonate-rich alkaline soils, uranyl readily forms stable aqueous carbonate complexes:

UO_2_^2+^ + 3CO_3_^2−^ ⇌ UO_2_(CO_3_)_3_^4−^

These complexes substantially increase uranium mobility and facilitate its transport toward plant roots [32,33].

In contrast, phosphate decreases uranium bioavailability by promoting the formation of poorly soluble uranium phosphate minerals:

3UO_2_^2+^ + 2PO_4_^3−^ → (UO_2_)_3_(PO_4_)_2_(s)

Furthermore, adsorption onto iron and manganese oxides and complexation with soil organic matter may further immobilize uranium, whereas changes in pH and redox potential can remobilize previously sorbed species [31,34,35].

Associated heavy metals undergo similar physicochemical transformations. Under acidic conditions, Cd^2+^, Zn^2+^, Ni^2+^, and Cu^2+^ mainly occur as hydrated ions and are therefore more readily available for plant uptake [34,36]. Increasing soil pH promotes adsorption or precipitation of these metals as sparingly soluble minerals. Representative precipitation reactions include:

Cd^2+^ + CO_3_^2−^ → CdCO_3_(s)

3Pb^2+^ + 2PO_4_^3−^ → Pb_3_(PO_4_)_2_(s)

Conversely, low-molecular-weight organic acids released by plant roots and rhizosphere microorganisms may form soluble metal–organic complexes, thereby enhancing metal mobility and bioavailability [37,38,39].

Chemical interactions among metals further regulate contaminant uptake. Because many metal ions possess similar ionic radii and charge, they compete for common membrane transport systems [40,41]. For example, Cd^2+^ competes with Zn^2+^ transport pathways, whereas arsenate (As(V)) enters root cells via phosphate transporters owing to its structural similarity to phosphate [41,42]. Likewise, cesium (Cs^+^) is absorbed primarily through potassium transport systems [43]. These competitive interactions influence not only contaminant accumulation but also the uptake of essential nutrients, thereby contributing to nutrient imbalance and physiological stress [44,45].

Overall, contaminant uptake is determined by the combined effects of chemical speciation, precipitation, sorption, complexation, and ion competition in the rhizosphere. Understanding these physicochemical mechanisms provides the basis for interpreting contaminant accumulation, toxicity, and plant adaptive responses discussed in the following sections.

## 4. Uptake of Radionuclides and Heavy Metals by Plants

Following their transformation into bioavailable forms, the subsequent migration of radionuclides and heavy metals depends on the ability of plants to absorb, transport, and accumulate these contaminants. The uptake of elements by plants is a complex physiological process involving root and foliar absorption, intracellular transport, translocation between organs, and subsequent accumulation in different tissues. The efficiency of these processes depends on both the physicochemical properties of the contaminants and plant-specific characteristics, including root architecture, transpiration rate, and the activity of membrane transport systems [14,46].

**Figure 3:**
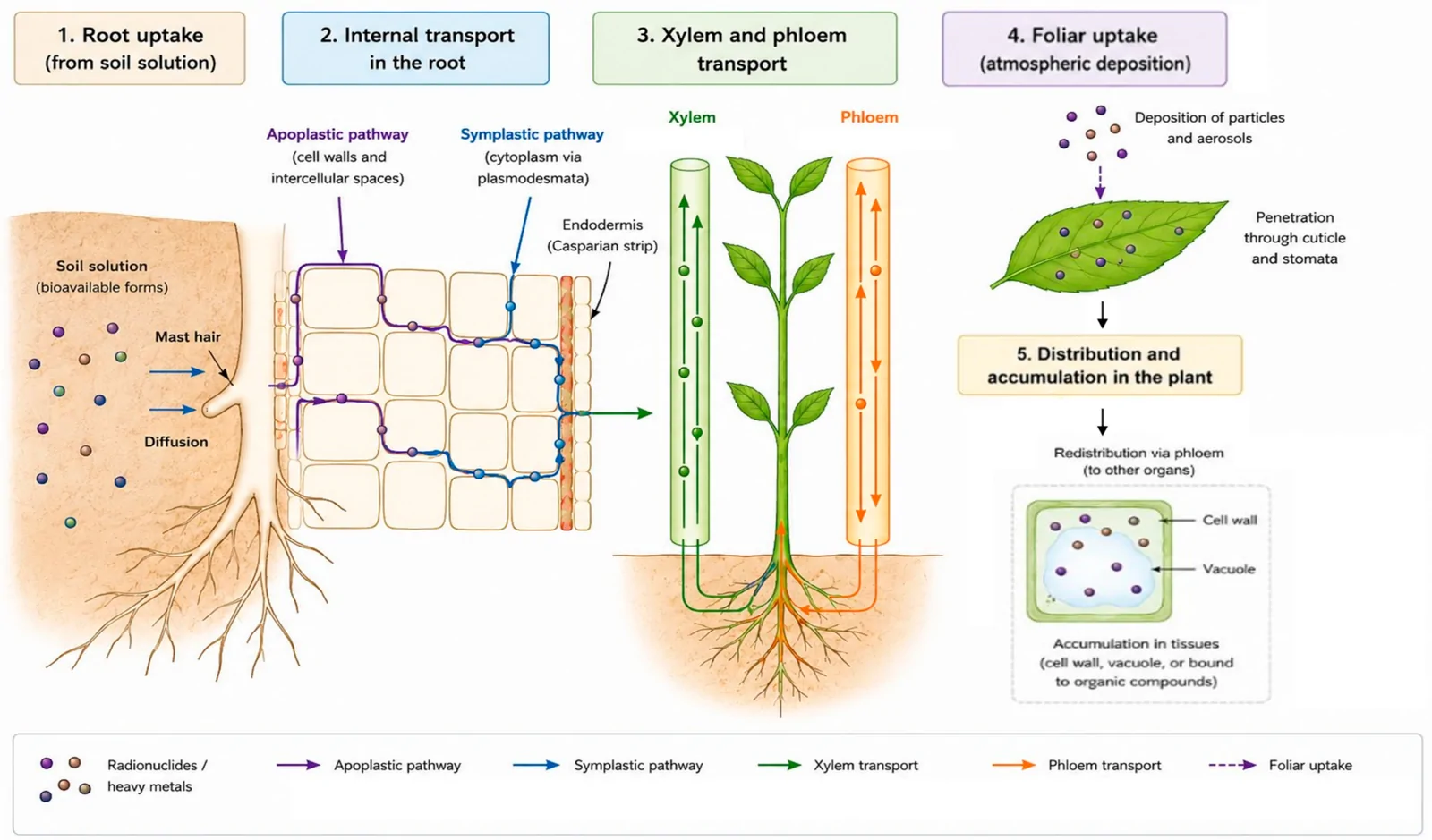
Schematic representation of the uptake, internal transport, translocation, and accumulation of radionuclides and associated heavy metals in plants through root and foliar pathways.

The main pathways involved in the uptake, internal transport, translocation, and accumulation of radionuclides and associated heavy metals in plants are summarized in Figure 3.

### 4.1. Root Uptake of Radionuclides and Heavy Metals

Root uptake represents the primary pathway by which radionuclides and heavy metals enter plants. Following mobilization within the rhizosphere, contaminants reach the root surface through mass flow and diffusion before entering epidermal cells and root hairs. Uptake is particularly intensive in the young root zone, which is characterized by high physiological activity and a large contact area with the surrounding soil solution [14].

Most radionuclides and heavy metals do not possess specific transport systems in plants but instead utilize membrane transporters responsible for the uptake of essential mineral nutrients. Owing to their chemical similarity to essential elements, toxic ions are able to enter root cells via existing transport pathways despite having no physiological function in plant metabolism [46,47].

Cadmium is one of the best-studied examples of this mechanism. It is predominantly taken up through transport systems responsible for zinc, iron, and calcium acquisition. Competition between cadmium and essential nutrients for common transporters largely determines its accumulation in plant tissues. Consequently, adequate zinc and iron nutrition can effectively reduce cadmium uptake by limiting its entry through these shared transport pathways [25,47,48].

Similarly, radioactive cesium (^137^Cs) is mainly absorbed through potassium transporters because of the close physicochemical similarity between Cs^+^ and K^+^ ions. Under potassium-deficient conditions, cesium accumulation increases substantially, whereas sufficient potassium supply significantly reduces ^137^Cs uptake and is regarded as one of the most effective agronomic measures for minimizing radiocesium accumulation in crops [43,49].

Natural radionuclides exhibit distinct uptake mechanisms. Radium is readily absorbed through calcium transport systems owing to its chemical similarity to calcium and can subsequently be translocated to aerial plant organs [35,36]. In contrast, uranium uptake is primarily controlled by its chemical speciation in the soil solution. Under aerobic conditions, uranium predominantly occurs as the UO_2_^2+^, whose uptake is mainly associated with phosphate transport systems and, possibly, calcium transporters. Consequently, uranium absorption is strongly influenced by its chemical speciation and the physicochemical conditions of the rhizosphere [46,50].

### 4.2. Internal Transport and Accumulation

Following entry into root tissues, radionuclides and heavy metals are transported via two principal pathways: the apoplastic and symplastic routes. The apoplastic pathway involves movement through cell walls and intercellular spaces without crossing plasma membranes, whereas the symplastic pathway proceeds through the cytoplasm of interconnected cells via plasmodesmata. At the endodermis, apoplastic transport is interrupted by the Casparian strip, forcing contaminants to cross the plasma membrane before entering the vascular tissues. This selective barrier plays an important role in regulating the entry of potentially toxic elements into the plant vascular system [47].

After crossing the endodermis, contaminants are loaded into the xylem and transported to aerial plant organs by the transpiration stream. The efficiency of xylem transport depends on transpiration rate, contaminant speciation, solubility, and the ability of metal ions to form complexes with organic ligands. During translocation, radionuclides and heavy metals are distributed among stems, leaves, and reproductive organs; however, a substantial proportion may remain immobilized within roots through adsorption to cell walls, sequestration in vacuoles, or complexation with organic compounds. Cadmium and cesium generally exhibit relatively high translocation efficiency, whereas uranium is predominantly retained within root tissues because of its strong binding to cell walls and limited mobility in the xylem [14,46,47].

### 4.3. Foliar Uptake

In addition to root uptake, foliar absorption may contribute to the accumulation of radionuclides and heavy metals, particularly in uranium mining regions where contaminated dust is continuously generated by waste rock dumps, tailings, and mining operations. Following atmospheric deposition, contaminants may either penetrate leaf tissues through the cuticle and stomata or remain adsorbed on the leaf surface, thereby increasing contaminant concentrations in aboveground plant organs [25,29].

The atmospheric pathway is particularly important for relatively immobile elements such as Pb, Th, and the radon decay products ^210^Pb and ^210^Po, for which atmospheric deposition may represent a significant source of accumulation in plant biomass. In contrast, uranium and most soluble heavy metals are predominantly absorbed through the root system from the soil solution [51].

Overall, the uptake and accumulation of radionuclides and heavy metals in plants are governed by their chemical speciation, bioavailability in soils, membrane transport activity, root and foliar uptake pathways, and the efficiency of intracellular and vascular transport. These processes ultimately determine contaminant distribution among plant organs and largely define the morphological, physiological, and biochemical responses of plants discussed in the following section.

Despite substantial progress in understanding contaminant uptake, considerable variability exists among published studies. Differences in uranium accumulation and translocation are largely attributed to plant species, contaminant speciation, soil physicochemical properties, and experimental conditions [1,46,47]. Moreover, many uptake mechanisms have been characterized under controlled laboratory or hydroponic conditions, whereas field investigations involve multiple interacting environmental factors that complicate direct comparisons [31]. Consequently, further long-term field studies are required to improve the ecological relevance of laboratory findings.

## 5. Morphological, Physiological, and Biochemical Biomarkers of Plants Exposed to Radionuclides and Heavy Metals in Uranium Mining Regions

Soil contamination in uranium mining areas and abandoned mine sites represents a complex environmental stressor resulting from the combined toxic effects of heavy metals (HMs) and ionizing radiation emitted by radionuclides. Plants growing under these extreme environmental conditions develop a wide range of adaptive responses that can serve as reliable biomarkers for environmental monitoring and ecological risk assessment [46]. Integrating morphological, physiological, and biochemical indicators provides a comprehensive evaluation of phytotoxicity and the phytoremediation potential of contaminated ecosystems [52,53].

**Figure 4:**
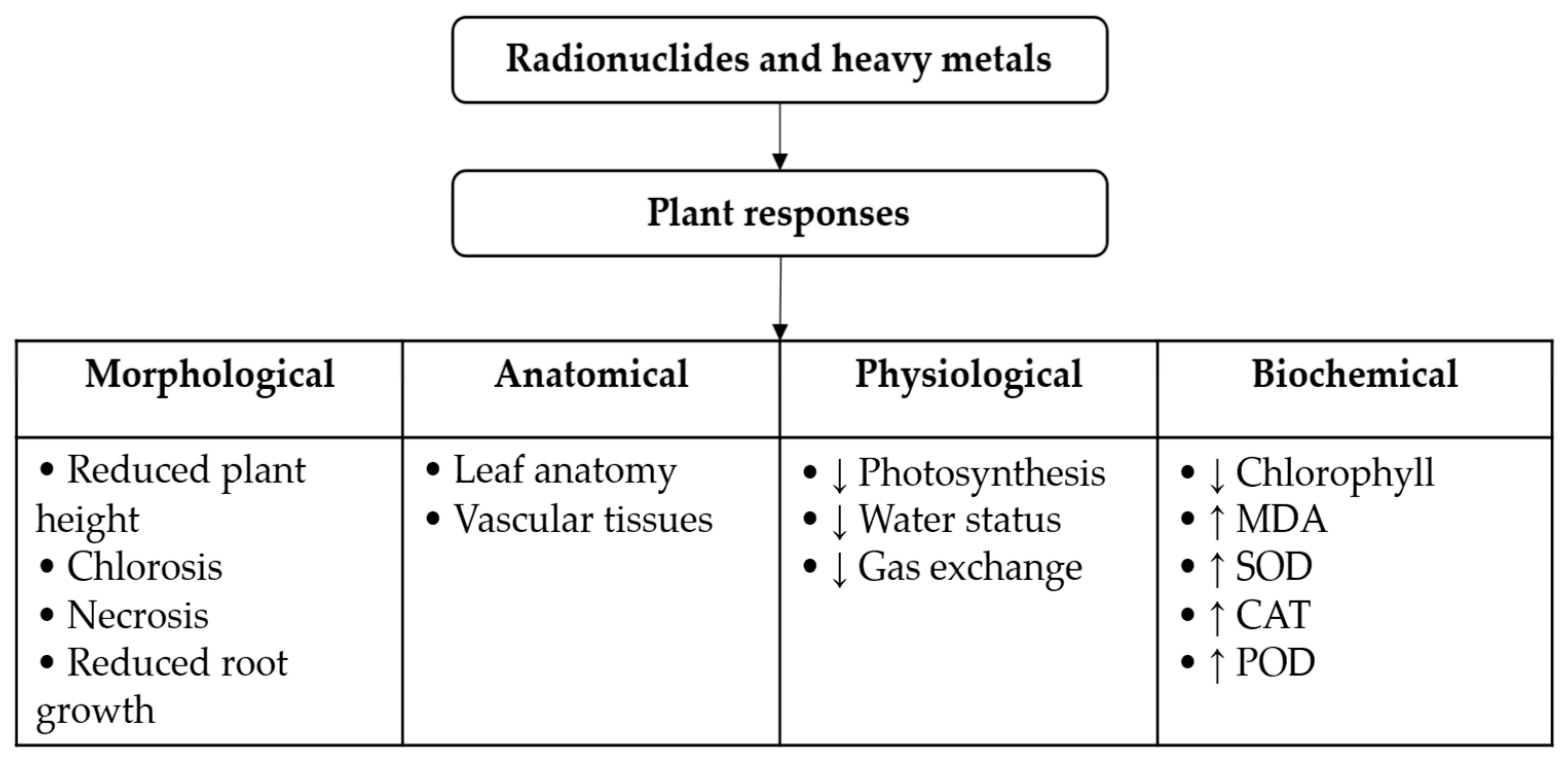
Main morphological, anatomical, physiological, and biochemical biomarkers of plant responses to radionuclides and heavy metals. Note: ↑ indicates an increase, while ↓ indicates a decrease.

An overview of the main morphological, anatomical, physiological, and biochemical biomarkers discussed in this section is presented in Figure 4.

### 5.1. Morphological and Anatomical Biomarkers as Primary Indicators of Environmental Stress

Morphological and anatomical characteristics are among the most readily observable indicators of plant responses to radionuclide and heavy metal contamination. Numerous studies conducted in uranium-contaminated environments have demonstrated that prolonged exposure to ionizing radiation and associated chemical pollutants induces structural modifications in plant tissues, including changes in cell size, cell wall thickness, vascular tissue organization, and overall plant morphology.

**Figure 5:**
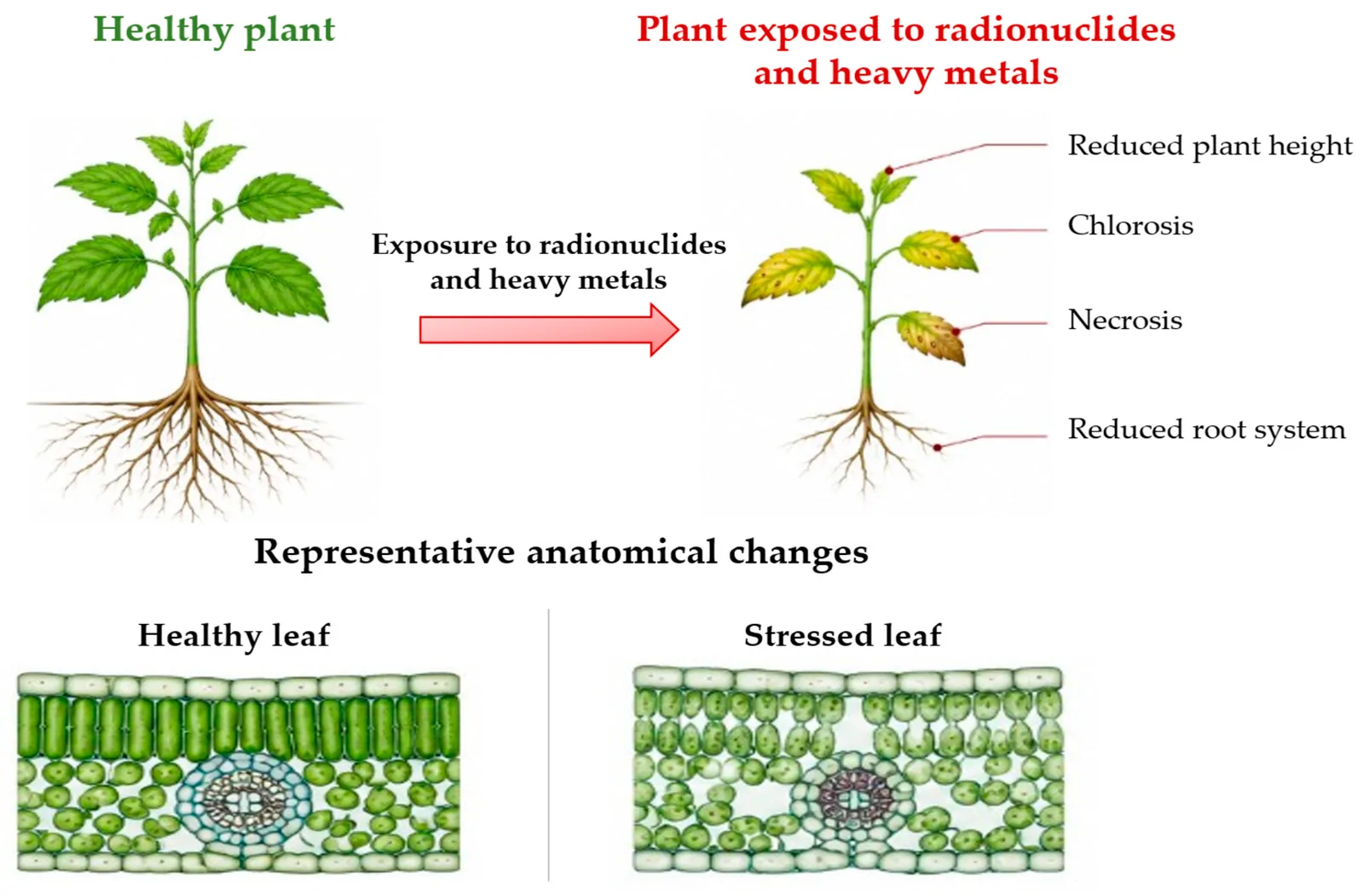
Representative morphological symptoms and anatomical changes in plants exposed to radionuclide and heavy metal stress.

Representative morphological symptoms and anatomical alterations commonly observed in plants exposed to radionuclides and heavy metals are illustrated in Figure 5. Typical morphological responses include reduced plant height, chlorosis, necrosis, and reduced root development, whereas representative anatomical changes are illustrated by leaf cross-sections showing alterations in tissue organization associated with long-term environmental stress.

Studies conducted at the Semipalatinsk Nuclear Test Site (Kazakhstan) demonstrated significant alterations in the morphological and anatomical characteristics of *Calamagrostis epigejos* (L.) Roth exposed to combined radionuclide and chemical contamination [54]. Similarly, investigations in Kalachi village (Akmola Region, Kazakhstan) revealed that chronic radiation exposure induces substantial anatomical and morphometric modifications in dominant plant species of different life forms [55]. These structural changes, particularly in vascular tissues and cellular architecture, were closely associated with radionuclide accumulation within plant organs, suggesting that anatomical traits can serve as sensitive indicators of long-term radioactive contamination.

Comparable observations have been reported in other uranium mining regions worldwide. In the Xiazhuang uranium ore field (Guangdong Province, China), dominant plant species, including *Castanopsis carlesii* (Hemsl.) Hayata, *Rhus chinensis* Mill., *Liriodendron chinense* (Hemsl.) Sarg., and *Sapium discolor* (Champ. ex Benth.) Müll.Arg., exhibited remarkable morphological stability despite accumulating uranium together with associated heavy metals (As, Cu, Pb, Mn, Mo, Zn, Cd, Co, and Ni). These findings indicate that prolonged exposure to contaminated environments promotes natural selection of highly tolerant plant populations capable of maintaining structural integrity under chronic metal and radionuclide stress [21].

Similarly, metabolomic investigations of wild plant species growing on abandoned metal mines in South Korea demonstrated that morphological resilience is closely associated with enhanced metabolic adaptation and phytoremediation potential [56]. Collectively, these studies from Kazakhstan, China, and South Korea demonstrate that, despite geographical differences, plants exposed to chronic radionuclide and heavy metal contamination exhibit common structural adaptations reflecting long-term environmental stress and can be effectively used as morphological biomarkers for ecological monitoring.

### 5.2. Physiological Biomarkers of Stress and Metabolic Disturbances

Physiological responses are among the earliest functional indicators of plant adaptation to radionuclide and heavy metal exposure. Uranium toxicity disrupts several fundamental physiological processes, including photosynthesis, water relations, mineral nutrition, and nitrogen metabolism, ultimately reducing plant growth and overall vitality. Recent studies have demonstrated that uranium also impairs endoplasmic reticulum protein homeostasis, leading to metabolic dysfunction and decreased cellular fitness [57].

Experimental studies on sunflower *Helianthus annuus* L. seedlings further demonstrated that uranium stress significantly alters physiological tolerance and the distribution of essential mineral elements, resulting in nutrient imbalance and impaired plant development [58]. These findings indicate that physiological parameters are highly sensitive indicators of uranium-induced metabolic disturbances and can reveal stress responses before visible structural damage occurs.

The disruption of plant metabolism begins with the interaction of heavy metals and radionuclides with the plasma membrane and membrane-associated proteins. Binding of metal ions alters membrane fluidity and permeability, resulting in electrolyte leakage, loss of ion homeostasis, and impaired transport of essential nutrients such as K^+^, Ca^2+^, Mg^2+^, and Fe^2+^ [59,60]. Consequently, water uptake, osmotic regulation, and nutrient acquisition become disrupted, leading to reduced photosynthetic efficiency, inhibition of respiration, impaired ATP production, and overall metabolic dysfunction. These physiological disturbances represent early events linking contaminant uptake to subsequent oxidative stress and biochemical responses [14,44,59].

Physiological adaptations have also been investigated in different ecosystem types. In freshwater ecosystems surrounding the uranium mill tailings pond at Jaduguda (India), aquatic macrophytes exhibited species-specific patterns of uranium uptake and accumulation, reflecting the increased bioavailability of radionuclides in aquatic environments compared with terrestrial systems [61]. Similarly, experiments conducted at the Nuclear Technology Development Centre (Brazil) identified *Epipremnum pinnatum* (L.) Engl. as a promising biomonitor of uranium and thorium due to its high capacity for element uptake, translocation, and accumulation under both uranium mine soil and hydroponic conditions [62].

Increasing attention has also been devoted to the combined effects of uranium and co-occurring heavy metals, which frequently coexist in uranium mining residues. Cadmium, one of the most common associated contaminants, markedly enhances uranium phytotoxicity. Experiments with *Raphanus sativus* L. demonstrated that simultaneous exposure to uranium and cadmium produced synergistic effects, resulting in stronger oxidative stress and greater inhibition of root growth than exposure to either contaminant alone [63]. Furthermore, uranium has been shown to suppress root metabolic activity, increase plasma membrane permeability, disrupt osmotic regulation, and alter reactive oxygen species (ROS) metabolism, thereby impairing multiple physiological functions essential for plant survival [64]. These findings highlight the importance of evaluating combined contaminant exposure when assessing plant responses in uranium mining environments.

Disruption of membrane integrity is therefore considered one of the earliest physiological responses to heavy metal toxicity, initiating a cascade of metabolic alterations that ultimately affect cellular homeostasis, energy metabolism, and plant growth [59,65].

### 5.3. Biochemical Biomarkers: Oxidative Stress and Antioxidant Defense

Heavy metal-induced oxidative stress is primarily initiated by disturbances in intracellular redox homeostasis. Metal ions interfere with electron transport chains in chloroplasts and mitochondria, promoting excessive generation of reactive oxygen species (ROS), such as superoxide radicals, hydrogen peroxide, and hydroxyl radicals. When ROS production exceeds the antioxidant capacity of plant cells, oxidative homeostasis is disrupted, resulting in lipid peroxidation, protein oxidation, DNA damage, and impairment of essential metabolic pathways [66,67].

Following the disruption of intracellular redox homeostasis, plants activate antioxidant defense systems to counteract excessive ROS accumulation. The primary enzymatic defense against oxidative stress includes superoxide dismutase (SOD), catalase (CAT), and peroxidase (POD), which cooperate to detoxify reactive oxygen species and protect cellular components from oxidative damage [66].

Superoxide dismutase (SOD) catalyzes the conversion of superoxide radicals into hydrogen peroxide, which is subsequently detoxified by catalase (CAT) and ascorbate peroxidase (APX). Peroxidases (PODs) and glutathione reductase (GR) further contribute to maintaining cellular redox homeostasis by eliminating peroxides and regenerating reduced glutathione. Under prolonged or severe heavy metal exposure, however, antioxidant defenses become overwhelmed, resulting in irreversible oxidative damage and metabolic dysfunction [68].

The phytoremediation potential of *Bidens pilosa* L. has been attributed, in part, to its efficient antioxidant defense system. Under uranium exposure, this species exhibited markedly increased activities of SOD, POD, and CAT, indicating an enhanced capacity to detoxify ROS while maintaining physiological performance in contaminated aquatic environments [69].

Comparable biochemical responses have been reported in heavy metal contaminated mining areas worldwide. In the abandoned Sidi Kamber mine (Skikda, Algeria), spectrophotometric analyses of plants growing on metal-contaminated soils revealed elevated levels of antioxidant activity together with increased synthesis of metal-binding proteins and low-molecular-weight chelating compounds, reflecting important mechanisms of metal tolerance and resistance [70]. Likewise, *Sesuvium portulacastrum* L. growing on contaminated soils in Jeddah (Saudi Arabia) maintained a highly reduced intracellular redox state, which was identified as a key physiological mechanism underlying heavy metal tolerance [71].

Collectively, these studies demonstrate that antioxidant enzyme activity, cellular redox status, and metal-chelating metabolites represent highly sensitive biochemical biomarkers of environmental stress and provide valuable information on plant tolerance mechanisms under chronic radionuclide and heavy metal exposure.

Taken together, morphological, physiological, and biochemical biomarkers provide complementary information on plant responses to radionuclide and heavy metal contamination. Morphological traits reflect the cumulative effects of long-term exposure, physiological parameters characterize the functional status of plants, whereas biochemical biomarkers serve as highly sensitive indicators of early cellular stress. The integration of these biomarkers enables a more comprehensive assessment of plant tolerance, environmental risk, and the phytoremediation potential of contaminated ecosystems.

For ease of comparison, Table 1 summarizes the principal bioindicator plant species investigated in uranium mining and other contaminated environments. The table presents the major contaminants together with the corresponding morphological, physiological, and biochemical responses reported in studies conducted across different countries.

The comparative analysis presented in Table 1 demonstrates that plant responses to uranium and heavy metal contamination occur at multiple levels of biological organization. Although the intensity of responses varies among species and environmental conditions, several common patterns can be identified.

At the morphological level, the most frequently reported responses include inhibition of root and shoot growth, reduction in biomass, alterations in leaf anatomy, and modifications of root architecture. Root tissues appear to be the primary target of uranium toxicity because they are the first organs exposed to contaminants and usually accumulate the highest concentrations of radionuclides and heavy metals.

Physiological responses are mainly associated with disturbances in photosynthesis, mineral nutrition, and water relations. Reductions in chlorophyll content, photosynthetic efficiency, and root activity were consistently observed across different plant species. At the same time, many bioindicator plants exhibited restricted root-to-shoot translocation of uranium, suggesting the presence of physiological barriers that reduce contaminant transport to aerial tissues.

Biochemical responses were found to be the most sensitive indicators of early stress. Almost all studies reported changes in antioxidant defense, including altered activities of superoxide dismutase (SOD), catalase (CAT), and peroxidase (POD), together with increased accumulation of malondialdehyde (MDA), proline, soluble proteins, or reactive oxygen species. These biomarkers provide valuable information on oxidative stress and the activation of protective mechanisms before visible morphological damage becomes apparent.

Overall, the published evidence indicates that no single biomarker is sufficient to characterize plant tolerance under radionuclide contamination. Instead, the integration of morphological, physiological, and biochemical indicators provides a more reliable assessment of plant health, contaminant toxicity, and phytoremediation potential. Species capable of maintaining physiological activity while restricting radionuclide translocation or efficiently accumulating contaminants in root tissues represent the most promising candidates for environmental biomonitoring and remediation of uranium-contaminated ecosystems.

### 5.4. Molecular Biomarkers of Plant Responses to Radionuclides and Heavy Metals

Although the preceding sections focus on morphological, anatomical, physiological, and biochemical biomarkers, recent studies have increasingly highlighted the importance of molecular biomarkers as highly sensitive indicators of early plant responses to uranium and associated heavy metals.

Molecular biomarkers provide early and sensitive indicators of plant responses to radionuclides and associated heavy metals, complementing morphological, physiological, and biochemical assessments. Unlike visible symptoms, molecular alterations occur at the gene and DNA levels before significant physiological impairment becomes apparent, making them valuable tools for environmental monitoring, ecological risk assessment, and evaluating plant adaptation in uranium mining regions [46,79].

One of the earliest molecular responses to contaminant exposure is the regulation of oxidative stress-related genes. Exposure to radionuclides and heavy metals induces changes in the expression of genes encoding antioxidant enzymes, including superoxide dismutase (SOD), catalase (CAT), and peroxidases (PODs), as well as genes involved in glutathione metabolism. These transcriptional changes reflect the activation of cellular defense pathways in response to excessive reactive oxygen species (ROS) and have been proposed as sensitive molecular biomarkers of contaminant-induced stress [76,80,81].

Genotoxicity represents another important class of molecular biomarkers. Radionuclides and heavy metals may induce DNA strand breaks, chromosomal aberrations, micronucleus formation, and mutations resulting from the combined effects of chemical toxicity and ionizing radiation [82,83,84,85]. In addition, molecular indicators of genomic instability, including DNA damage and chromosomal alterations, have been successfully applied to assess plant responses under both laboratory and field conditions [83,84,85].

Recent advances in transcriptomics, proteomics, metabolomics, and ionomics have considerably expanded the range of molecular biomarkers available for evaluating plant responses to environmental contamination [86]. These high-throughput approaches enable the identification of stress-responsive genes, proteins, metabolites, and regulatory pathways associated with radionuclide and heavy metal exposure, thereby improving the mechanistic understanding of plant tolerance and supporting the development of effective biomonitoring and phytoremediation strategies.

Although numerous biomarkers have been proposed to assess plant responses to radionuclides and associated heavy metals, each biomarker group has inherent limitations. Morphological biomarkers are the most suitable for long-term environmental monitoring because they integrate the cumulative effects of prolonged contaminant exposure, although they generally reflect relatively late responses to stress. Physiological and biochemical biomarkers provide earlier evidence of functional impairment and are therefore valuable for detecting ongoing stress before visible symptoms develop. Molecular biomarkers provide highly sensitive indicators of early cellular responses; however, their application is constrained by species-specific gene expression, environmental variability, and the need for specialized analytical techniques [44,86]. Therefore, integrating morphological, physiological, biochemical, and molecular biomarkers provides the most comprehensive assessment of plant responses and improves the reliability of environmental monitoring and ecological risk assessment.

### 5.5. Plant Resistance Mechanisms to Radionuclide and Heavy Metal Stress

Plant survival in uranium-contaminated environments depends on a coordinated network of resistance mechanisms that minimize contaminant toxicity while maintaining cellular homeostasis. These mechanisms operate at multiple levels of biological organization and include restriction of contaminant uptake and translocation, intracellular detoxification, activation of antioxidant defense systems, metabolic reprogramming, and structural adaptation. Rather than acting independently, these responses function in an integrated manner to enhance plant tolerance under prolonged exposure to radionuclides and associated heavy metals [44,59].

One of the earliest resistance strategies is the regulation of contaminant uptake and internal transport. Many plant species restrict the movement of radionuclides and heavy metals from roots to shoots by retaining contaminants within root tissues. Adsorption to cell walls, binding to negatively charged polysaccharides, and sequestration within root vacuoles reduce xylem loading and limit contaminant transport to photosynthetically active organs. Uranium is particularly prone to root retention because uranyl ions readily interact with phosphate-containing cell wall components, resulting in low translocation to aerial tissues. Such exclusion mechanisms reduce toxicity and contribute to the phytostabilization capacity of many uranium-tolerant species [1,47].

Following cellular entry, plants activate detoxification mechanisms that decrease the chemical reactivity of toxic ions. Low-molecular-weight ligands, including phytochelatins, metallothioneins, organic acids, and glutathione, chelate heavy metals and radionuclides, thereby reducing their interaction with proteins, nucleic acids, and biological membranes. The resulting metal–ligand complexes are subsequently transported into vacuoles, where contaminants are compartmentalized and isolated from metabolically active cellular compartments. Vacuolar sequestration is therefore considered one of the principal mechanisms underlying long-term heavy metal tolerance [65,87,88].

The maintenance of redox homeostasis represents another essential component of plant resistance. Excessive accumulation of reactive oxygen species during heavy metal exposure triggers the activation of antioxidant defense systems, including superoxide dismutase (SOD), catalase (CAT), ascorbate peroxidase (APX), peroxidases (PODs), and glutathione reductase (GR), together with non-enzymatic antioxidants such as glutathione, ascorbate, proline, phenolic compounds, and flavonoids. These antioxidant systems limit oxidative damage, stabilize cellular membranes, protect photosynthetic machinery, and maintain metabolic activity under stress conditions [66,68].

Long-term adaptation also involves physiological and morphological adjustments that improve plant performance in contaminated environments. Alterations in root architecture increase the capacity for selective nutrient acquisition while reducing contaminant uptake. Plants additionally regulate water relations, nutrient transport, osmotic balance, and photosynthetic activity to compensate for heavy metal-induced metabolic disturbances. In chronically contaminated uranium mining regions, these adaptive responses may ultimately lead to the establishment of naturally tolerant plant populations capable of surviving and reproducing under persistent radionuclide and heavy metal stress [44,46,47].

Overall, plant resistance to heavy metals and radionuclides results from the coordinated interaction of exclusion, detoxification, antioxidant protection, and physiological adaptation mechanisms. Understanding these integrated responses is essential for identifying suitable bioindicator species and selecting plants with high potential for phytostabilization and phytoextraction in uranium-contaminated ecosystems.

## 6. Phytoremediation of Uranium-Contaminated Mining Areas

Phytoremediation has emerged as an environmentally sustainable and cost-effective strategy for restoring soils contaminated by uranium mining and processing activities. Uranium, as a chemically toxic and radioactive element, is released into the environment through mining operations, milling processes, tailings disposal, and in situ leaching, posing long-term risks to ecosystems and human health [1,31]. Unlike conventional remediation technologies, such as soil excavation or chemical washing, which are often expensive and may cause secondary environmental disturbance, phytoremediation relies on the natural physiological processes of plants to immobilize, accumulate, or remove contaminants from the environment [31,89].

**Figure 6:**
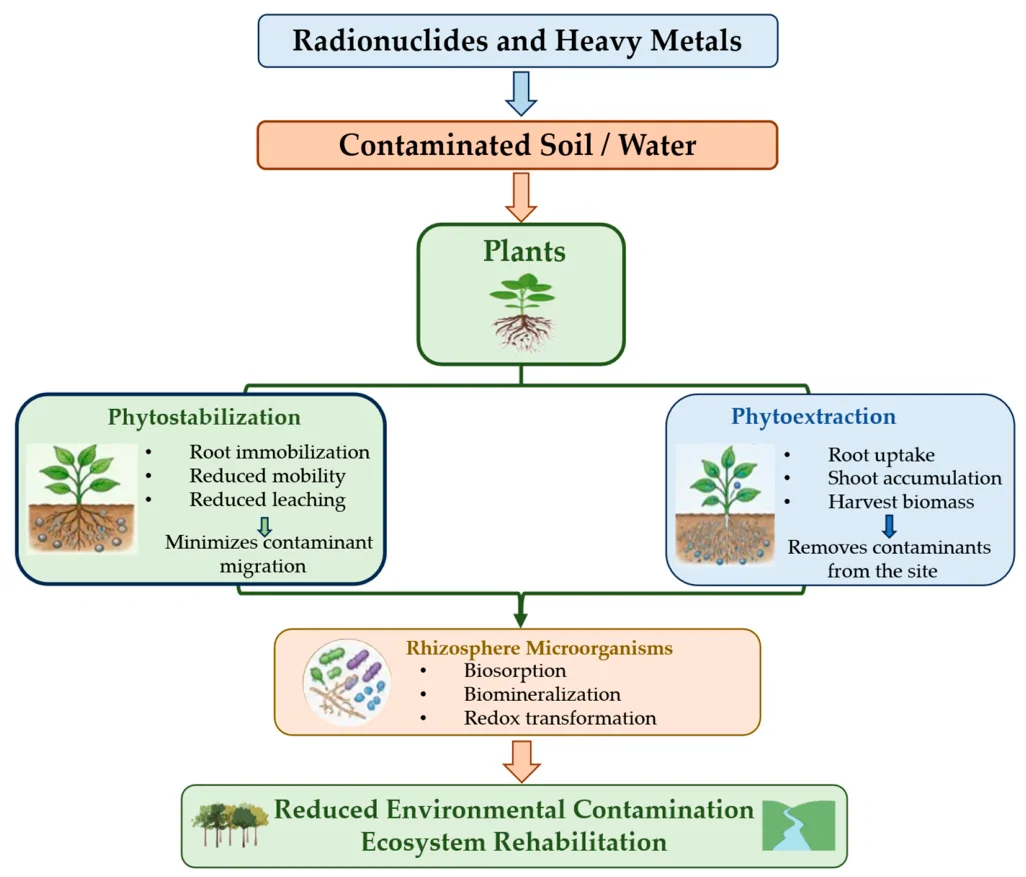
Schematic representation of the main phytoremediation processes in uranium- and heavy metal-contaminated environments.

The two principal phytoremediation mechanisms applied to uranium-contaminated soils are phytostabilization and phytoextraction. Phytostabilization aims to reduce uranium mobility by immobilizing it within the rhizosphere through root adsorption, precipitation, or complexation with root exudates, thereby minimizing its migration into groundwater and surrounding ecosystems [1,4]. In contrast, phytoextraction involves the uptake of uranium by plant roots followed by its translocation to aboveground biomass, which can subsequently be harvested and safely disposed of or processed for metal recovery [4]. The principal phytoremediation processes discussed in this section are summarized in Figure 6.

The efficiency of phytoremediation largely depends on plant species, soil physicochemical properties, uranium speciation, and environmental conditions. Perennial ryegrass (*Lolium perenne* L.), for example, accumulates uranium predominantly within root cell walls, reducing its phytotoxic effects while maintaining relatively high uptake efficiency [90]. The application of biodegradable chelating agents, particularly citric acid, can significantly increase uranium bioavailability and improve phytoextraction efficiency. However, excessive chelator application may enhance uranium leaching into deeper soil layers and groundwater, requiring careful optimization of treatment conditions [90].

Phytoremediation is particularly important in uranium mining regions, where waste rock dumps and tailings repositories remain long-term sources of radioactive and chemical contamination [2]. Besides uranium, these sites commonly contain associated toxic elements such as arsenic, molybdenum, vanadium, and other heavy metals, requiring integrated remediation approaches capable of addressing multi-element contamination [2].

Recent studies demonstrate that remediation efficiency can be considerably improved by combining multiple plant species within artificially constructed plant communities. Such systems promote complementary nutrient utilization, increase rhizosphere organic acid production, improve soil structure, and enhance contaminant uptake through synergistic plant–plant interactions [91]. Equally important is the role of rhizosphere microorganisms, which regulate uranium bioavailability through redox transformations, biosorption, biomineralization, and complexation processes. Consequently, interactions between plants and rhizosphere microbial communities are increasingly recognized as one of the key factors determining phytoremediation success [92].

To facilitate comparison of current phytoremediation approaches, Table 2 summarizes representative plant species and remediation strategies investigated for uranium-contaminated soils, together with their principal remediation mechanisms and reported applications.

The studies summarized in Table 2 demonstrate that the efficiency of phytoremediation depends on plant species, remediation strategy, and site-specific conditions. Phytoextraction is suitable for removing uranium from contaminated soils, whereas phytostabilization is more effective for limiting the mobility of uranium and associated heavy metals. Recent approaches combining soil amendments and plant–microbe interactions further improve remediation efficiency and represent promising strategies for the restoration of uranium-contaminated ecosystems.

Despite the promising potential of phytoremediation, its practical application in uranium mining regions remains constrained by several methodological and environmental limitations. Most available studies have been conducted under laboratory or greenhouse conditions, whereas relatively few have evaluated remediation efficiency under long-term field conditions [1,31,46]. Consequently, the effectiveness of phytoremediation reported in the literature varies considerably owing to differences in soil physicochemical properties, uranium speciation, climatic conditions, plant species, and the presence of co-contaminants. These factors complicate direct comparisons among studies and limit the transferability of laboratory findings to field-scale applications. Furthermore, the management of contaminated plant biomass after harvesting remains an important challenge for the practical implementation of phytoextraction. Future research should therefore prioritize long-term field validation using native plant species, standardized evaluation protocols, and a better understanding of plant–microbe interactions to improve the reliability, efficiency, and large-scale application of phytoremediation technologies.

## 7. Discussion

The reviewed studies demonstrate that vegetation in uranium mining regions is exposed to complex environmental stress resulting from the combined effects of radionuclides and associated heavy metals. Plant responses are determined not only by contaminant concentrations but also by their chemical speciation, bioavailability, and environmental factors regulating contaminant mobility. Consequently, reliable ecological risk assessment requires an integrated evaluation of contaminant behavior rather than relying solely on total elemental concentrations.

A consistent finding across the literature is that plant responses occur at multiple levels of biological organization. Morphological alterations generally reflect long-term cumulative exposure, physiological responses indicate disturbances in photosynthesis, nutrient acquisition, and water relations, whereas biochemical biomarkers provide the earliest evidence of oxidative stress through changes in antioxidant enzyme activity and related metabolites. The integration of these indicators therefore offers a more comprehensive and reliable assessment of plant health and environmental contamination than any single biomarker alone.

The reviewed studies also reveal substantial interspecific differences in uranium uptake, translocation, and tolerance. Species capable of restricting uranium transport to aboveground organs are particularly valuable for phytostabilization, whereas species with efficient uptake and translocation are more suitable for phytoextraction. These differences indicate that successful remediation strategies should be based on site-specific environmental conditions and the biological characteristics of the selected plant species.

Recent advances further demonstrate that phytoremediation efficiency can be enhanced by combining tolerant plant species with biodegradable chelating agents, biochar amendments, and beneficial rhizosphere microorganisms. Such integrated approaches improve contaminant immobilization or extraction while simultaneously promoting soil restoration and ecosystem recovery. However, most available evidence is derived from controlled laboratory or greenhouse experiments, whereas long-term field validation remains limited.

The evidence summarized in the phytoremediation section further indicates that phytostabilization and phytoextraction should be regarded as complementary rather than competing remediation strategies. Their successful implementation depends on contaminant bioavailability, soil physicochemical properties, the selection of appropriate plant species, and interactions with rhizosphere microorganisms. Integrating biomarker-based ecological assessment with phytoremediation planning may therefore improve the long-term restoration and management of uranium-contaminated ecosystems.

Despite Kazakhstan being one of the world’s leading uranium-producing countries, relatively few studies have investigated the responses of native vegetation to uranium contamination. Existing research has mainly focused on morphological and anatomical changes, while comprehensive studies integrating physiological, biochemical, and phytoremediation aspects remain scarce. Expanding research on native plant species under field conditions would improve ecological monitoring and support the development of effective restoration strategies for uranium mining landscapes in Kazakhstan.

Future research should therefore focus on identifying native uranium-tolerant species, clarifying plant–microbiome interactions, and evaluating integrated phytoremediation technologies under long-term field conditions to simultaneously address radionuclides and associated heavy metals.

## 8. Conclusions

Uranium mining and processing create long-term environmental contamination by radionuclides and associated heavy metals, posing persistent challenges to ecosystem integrity and environmental sustainability. The evidence reviewed demonstrates that the ecological impact of these contaminants depends not only on their concentrations but also on their chemical speciation, mobility, and bioavailability, which govern their transfer through the soil–plant system. Plants respond to chronic exposure through coordinated morphological, physiological, biochemical, and molecular adaptations that reflect different levels of biological organization. The integration of these complementary biomarkers provides a more reliable assessment of plant health, contaminant toxicity, and ecosystem condition than individual indicators alone, making them valuable tools for ecological monitoring and environmental risk assessment.

Substantial evidence has established the main factors controlling radionuclide and heavy metal bioavailability, plant uptake and accumulation, as well as the principal morphological, physiological, biochemical, and molecular responses of plants to contamination. Considerable progress has also been made in understanding the mechanisms and applications of phytostabilization and phytoextraction for the rehabilitation of uranium-contaminated ecosystems.

This review highlights phytoremediation as a promising, environmentally sustainable approach for the rehabilitation of uranium-contaminated ecosystems. Current evidence indicates that the success of phytostabilization and phytoextraction depends on the selection of appropriate plant species, contaminant bioavailability, soil characteristics, and interactions with rhizosphere microorganisms, while soil amendments may further enhance remediation efficiency. Despite substantial progress, important knowledge gaps remain, particularly regarding long-term field validation, integrated multi-biomarker approaches, plant–microbiome interactions, and the identification of native uranium-tolerant species in major uranium-producing regions such as Kazakhstan. Addressing these challenges will strengthen ecological monitoring, improve environmental risk assessment, and support the development of effective, science-based restoration strategies for uranium-affected landscapes.

## Figures and Tables

**Table 1:** Comparative Overview of Morphological, Physiological, and Biochemical Responses of Plants Exposed to Radionuclides and Heavy Metals in Field and Experimental Studies.

Country	Plant Species	Contaminant (Radionuclide/Heavy Metal)	Morphological Responses	Physiological Responses	Biochemical Responses	Reference
Kazakhstan	*C. epigejos*	^137^Cs, ^90^Sr, U, Pb, Cd, Al, Ni and other toxic elements	Alterations in leaf and stem anatomy; changes in mesophyll thickness, epidermis thickness and vascular bundle structure	Not evaluated	Not evaluated	[54]
Kazakhstan	*Festuca valesiaca* Schleich. ex Gaudin, *Galatella linosyris* (L.) Rchb.f.	U, Th and associated heavy metals	Reduced leaf blade thickness; changes in epidermis thickness, vascular bundle size, cortex structure and stem anatomy	Not evaluated	Not evaluated	[55]
China	*C. carlesii*, *R. chinensis*	U, As, Cu, Pb, Zn, Cd	No pronounced morphological damage; high tolerance under long-term contamination.	Species-specific accumulation of U and heavy metals; high bioaccumulation of Cd, Mn and Cu (*S. discolor*, *L. chinense*, *C. carlesii*); changes in chlorophyll (pigment) content under U exposure; *C. carlesii* maintained stable photosynthetic pigment characteristics	Species-specific biochemical responses to uranium stress; *R. chinensis* exhibited sensitive biochemical responses, whereas *L. chinense* showed weak biochemical responses	[21]
China	*R. sativus*	U, Cd	Reduced total and root biomass (root biomass decreased by 11.9–63.8%); inhibition of seedling growth; increased root cell death; decreased root-to-shoot ratio.	Increased uranium accumulation in roots (1.2-fold under combined U + Cd exposure); impaired root activity; increased plasma membrane permeability; disturbance of root physiological functions.	Increased O_2_^−^ and H_2_O_2_ production; increased malondialdehyde (MDA), proline and soluble protein contents; decreased activities of SOD, CAT and POD under combined U + Cd stress, indicating enhanced oxidative stress.	[63]
China	*B. pilosa*	U	No obvious morphological injury; high uranium accumulation in roots, stems and leaves (up to 1537.75 mg kg^−1^ DW).	Photosynthetic efficiency (Fv/Fm) decreased with increasing U concentration; photochemical efficiency (ΦPSII, qP, qL) declined; uranium accumulated in both roots and shoots, indicating efficient translocation.	SOD activity decreased, whereas POD and CAT activities initially increased (≤240 μM U) and then declined at higher U concentrations; soluble sugar increased at low U concentrations but decreased at high concentrations; soluble protein increased at low U concentrations and decreased under severe U stress.	[69]
Russia	*Vicia cracca* L.	^226^Ra, ^238^U, ^232^Th	Not evaluated	Decreased seed germination and sprout survival; reduced reproductive capacity; increased chromosome aberration frequency with increasing absorbed dose	Not evaluated	[72]
Algeria	*Cistus monspeliensis* L., *Reichardia bucephalophora* (Willd.) B.Nord., *Verbascum sinuatum* L.	Zn, Pb, Cd, Cu	Metal tolerance and species-specific distribution between roots and shoots	Species-specific accumulation and translocation of heavy metals; decreased chlorophyll content under high metal concentrations	Increased proline, soluble protein and soluble sugar contents	[70]
India	*Pisum sativum* L.	U	Reduced root growth	Decreased chlorophyll and carotenoid contents	Increased MDA; decreased SOD and GR activities	[73]
India	*H. annuus*	Uranium tailings	Reduced seed germination, leaf area, shoot length, root length, fresh and dry biomass; leaf yellowing.	Decreased tolerance index (TI); increased grade of growth inhibition (GGI).	Decreased chlorophyll a, chlorophyll b and total chlorophyll; increased soluble protein content.	[74]
France	*Arabidopsis* *thaliana* (L.) Heynh.	U	Inhibition of primary root growth at toxic U concentrations; increased lateral root formation; altered root architecture; reduced cell viability in the root apex.	Phosphate depletion; redistribution of Fe in root tissues; altered mitotic activity; disturbance of root meristem function.	Increased ROS and NO production; enhanced callose and lignin deposition indicating activation of defense responses.	[75]
Belgium	*Phaseolus vulgaris* L.	U	Leaf chlorosis and root yellowing at 1000 μM U; no significant reduction in plant growth, root biomass, shoot biomass, leaf biomass or plant length at lower U concentrations.	Strong uranium accumulation in roots with limited root-to-shoot translocation; root/shoot transfer decreased with increasing U concentration.	Oxidative stress induced by U; stimulation of antioxidant enzymes (GPOD, SPOD, GLUR, ICDH, G6PDH) at low–moderate U, inhibition at 1000 μM; increased reduced glutathione (GSH) and GSH/GSSG ratio at ≤100 μM U, depletion of GSH at 1000 μM; severe DNA damage in roots at 1000 μM U.	[76]
Belgium	*A. thaliana*	U	Reduced root and leaf fresh weight; inhibition of root growth	Strong U accumulation in roots; limited root-to-shoot translocation; decreased Fe content in leaves	Increased APX and GR activity in roots; transient increase in AsA and GSH in leaves at low U concentrations followed by collapse of antioxidant defence at high U concentrations; altered expression of antioxidant genes (CSD, FSD, CAT, GSH1/GSH2)	[77]
Portugal	*Cistus ladanifer* L., *Helichrysum stoechas* (L.) DC., *Hypochaeris* *radicata* L., *Juncus* *squarrosus* L., *Lemna minor* L.	U	Not evaluated	Species-specific uranium uptake and accumulation; preferential accumulation in roots/rhizomes; limited translocation to aerial parts due to physiological barriers; several species identified as potential bioindicators of uranium contamination.	Not evaluated	[78]

**Table 2:** Representative Plant Species and Phytoremediation Strategies for Uranium and Associated Heavy Metal-Contaminated Soils.

Plant Species/Remediation Strategy	Primary Phytoremediation Mechanism	Efficiency and Application Characteristics	Reference
Biochar amendment	Assisted phytostabilization/ uranium immobilization	Reduced soil uranium bioavailability by 58.9% and shoot uranium accumulation by 39.7%; improved soil microenvironment, promoted plant growth, alleviated oxidative stress, and immobilized uranium through surface complexation, reduction, ion exchange, and physical adsorption.	[3]
*L. perenne*	Phytoextraction (mainly in roots)	Predominant uranium accumulation in root cell walls; effective uranium uptake from contaminated soil.	[90]
Artificial plant community (*Salix* sp. + *Paspalum scrobiculatum* L. + *Macleaya cordata* (Willd.) R.Br.)	Enhanced phytoextraction	Mixed plant community increased uranium bioaccumulation (up to 146%), transfer factor and plant biomass compared with monocultures through rhizosphere interactions and increased organic acid production.	[91]
*Brassica juncea* L. *+ slow-release citric acid*	Enhanced phytoextraction	Slow-release citric acid increased uranium uptake, enhanced phytoextraction efficiency, and reduced uranium leaching risk.	[93]
*Triticum aestivum* L.; *H. annuus*	Phytoextraction	*H. annuus* showed higher uranium uptake from Saghand mine soil, whereas *T. aestivum* was more effective in Bandar Abbas soil without or at low citric acid concentrations. Increasing citric acid concentration enhanced uranium uptake by sunflower.	[94]
*Pteris vittata* L.	Enhanced phytoextraction	Decreased As bioavailability, enhanced As and Pb phytoextraction, improved soil quality, and promoted rhizosphere bacterial diversity.	[95]
*Sedum alfredii* Hance	Phytoextraction	Hyperaccumulator of Cd and Zn; exhibits high metal uptake, efficient root-to-shoot translocation, and strong hypertolerance, making it a promising species for phytoremediation of heavy metal-contaminated soils.	[96]
*Salix viminalis* L.	Phytoextraction	High-biomass willow capable of extracting Cd and Zn under field conditions; effective long-term removal of Cd and Zn from contaminated soils.	[97]
*Noccaea caerulescens* (J.Presl & C.Presl) F.K.Mey. *(Thlaspi caerulescens)*	Phytoextraction	Hyperaccumulator of Cd and Zn with exceptional metal tolerance and high shoot accumulation. Considered one of the most effective plant species for Cd phytoextraction from contaminated soils and a model species for phytoremediation research.	[98]
*Chrysopogon zizanioides* (L.) Roberty, (Vetiver grass)	Phytostabilization/ Phytoextraction	Deep-rooted perennial grass with high tolerance to heavy metal-contaminated soils. Widely used for rehabilitation of open-pit mines and mine tailings, erosion control, and stabilization of contaminated soils. Also applied in the treatment of heavy metal-contaminated wastewater using constructed wetlands.	[99]

## Data Availability

The data presented in this study are available upon request from the corresponding authors, Madina Kairullova and Danara Ibrayeva.
